# Supplementary material for: A comparative study between Near-Infrared (NIR) spectrometer and High-Performance Liquid Chromatography (HPLC) on the sensitivity and specificity
Source: PLoS One. 2025 Mar 25;20(3):e0319523. doi: 10.1371/journal.pone.0319523 (PMC11936202; doi:10.1371/journal.pone.0319523)
Supplement: S1 Table — (DOCX) [file pone.0319523.s001.docx]

**S1 Table. Analytical Parameters for Linearity, Correlation, and Detection Limits of Compounds by High-Performance Liquid Chromatography.**

| **Compound** | **Linearity Range (µg/mL)** | **Linearity** | **Correlation (r^2^)** | **LOD (µg/mL)** | **LOQ (µg/mL)** |
| --- | --- | --- | --- | --- | --- |
| Amiloride HCl | 2 - 100 | y=33.83343x-7.55016 | 0.99988 | 1.9 | 6.27 |
| Artemether | N/A | N/A | N/A | 100 | 330 |
| Artemisinin | 100 - 1000 | y=0.822018x-0.9991509 | 0.99997 | 50 | 100 |
| Caffeine | 0.12 - 12 | y=58.63687x+0.656616 | 0.99998 | 0.07 | 0.231 |
| Ciprofloxacin | 2 - 200 | y=68.85823x+2.985 | 0.99999 | 0.8 | 2.64 |
| Diphenhydramine HCl | 0.5 - 10 | y=42.29088x+11.50653 | 0.99923 | 0.5 | 1.65 |
| Hydrochlorothiazide | 20 - 1000 | y=9.08837x-89.63894 | 0.99949 | 10 | 33 |
| Lumefantrine | N/A | N/A | N/A | 1.2 | 3.96 |
| Methyldopa | 10 - 200 | y=6.20367x-5.66193 | 0.99981 | 4.9 | 16.17 |
| Metronidazole | 5 - 200 | y=7.36814x-0.810197 | 0.99996 | 2.2 | 7.26 |
| Nifedipine | 5 - 200 | y=32.68289x-15.47216 | 0.99994 | 2.5 | 8.25 |
| Ofloxacin | 2 - 100 | y=41.08872x-13.16695 | 0.99998 | 0.8 | 2.64 |
| Paracetamol | 1 - 200 | y=18.25010x+4.17424 | 0.99997 | 1.7 | 5.61 |
| Paracetamol | 2 - 200 | y=36.65655x+31.27483 | 0.99996 | 2.1 | 6.93 |
| Piperaquine | 20 - 500 | y=23.42863x-241.46747 | 0.99914 | 20 | 66 |
| Pyrantel Pamoate | 10 - 200 | y=4.010623x-4.19306 | 0.99988 | 4 | 13.2 |
| Pyrimethamine | 1.25 - 40 | y=104.39893x-13.93893 | 0.99997 | 0.4 | 1.32 |
| Sulfadoxine | 25 - 800 | y=21.96004x-17.55829 | 0.99997 | 7.3 | 24.09 |
| Tinidazole | 6 - 300 | y=16.10292x+2.11092 | 0.99996 | 3.4 | 11.22 |
