# Supplementary material for: A comparative study between Near-Infrared (NIR) spectrometer and High-Performance Liquid Chromatography (HPLC) on the sensitivity and specificity
Source: PLoS One. 2025 Mar 25;20(3):e0319523. doi: 10.1371/journal.pone.0319523 (PMC11936202; doi:10.1371/journal.pone.0319523)
Supplement: S2 Table — (DOCX) [file pone.0319523.s002.docx]

**S2 Table. Sample Preparation and HPLC Parameters for Compound Analysis**

| **Compound** | **Sample Preparation** | **HPLC Parameters** |
| --- | --- | --- |
| Paracetamol and Caffeine | *Standard Preparation:* Dissolve paracetamol and caffeine standard in extraction solvent. Sonicate for 10mins. Prepare calibration standards in mobile phase. *Sample Preparation:* For samples containing just Paracetamol, pulverize 20 tablets and weigh an equivalent of 100mg paracetamol and 6mg of Caffeine into a 100mL flask. Add 75mL methanol/water (1:3) and sonicate for 10mins and make up to the mark with same extraction solvent. Transfer 1mL of the solution to a 10mL flask and make up to the mark with extraction solution. Filter with a 0.45µm syringe filter and transfer filtrate to vial for HPLC analysis. | MP: Water/Methanol (80:20) SP: Waters Xbridge C18 (100 x 4.6mm I.D, 5um) FR: 1.0mL/min, CT.: 35ᵒC, Inj. vol.: 20µL, λ: 275nm |
| Paracetamol and Diphenhydramine | *Standard Preparation:* Dissolve paracetamol and diphenhydramine (DPH) standard in methanol/glacial acetic acid (9.5:0.5). Sonicate for 10mins. Prepare calibration standards in mobile phase. *Sample Preparation:* Pulverize 20 tablets and weigh an equivalent to 100mg of PCM and 5mg of DPH into a 100mL flask. Add 75mL extraction solvent. Sonicate for 10mins and make up to the mark with same extraction solvent. Transfer 1mL of the solution to a 10mL flask and make up to the mark with extraction solution. Filter with a 0.45µm syringe filter and transfer filtrate to vial for HPLC analysis. | MP: 0.1%v/v trifluoroacetic acid in water/acetonitrile (80:20) SP: Phenomenex Luna C18 (250 x 4.6mm I.D, 5um) FR: 1.0mL/min, CT.: 35ᵒC, Inj. vol.: 20µL, λ: 220nm |
| Amiloride HCl and Hydrochlorothiazide | *Amiloride Standard Preparation:* Prepare 1mg/mL of Amiloride Hydrochloride in Methanol. Hydrochlorothiazide and Amiloride Standard Mix: Transfer 10mL of 1mg/mL Amiloride HCl standard solution into a 100mL flask containing 100mg of Hydrochlorothiazide and 20mL of Methanol. Add 4mL of 1N HCl and dilute to volume with water. *Sample Preparation:* Pulverize 20 tablets and weigh an equivalent to 50mg Hydrochlorothiazide and 5mg of Amiloride HCl into a 100mL flask. Add 15mL of methanol and 2mL of 1N HCl, sonicate for 10mins and make up to the mark with water. Sonicate for another 10mins. Filter with a 0.45µm syringe filter and transfer filtrate to vial for HPLC analysis. | MP: 0.1%v/v trifluoroacetic acid in water/acetonitrile (80:20) SP: Phenomenex Luna C18 (250 x 4.6mm I.D, 5um) FR: 1.0mL/min, CT.: 35ᵒC, Inj. vol.: 10µL, λ: 286nm |
| Artemether and Lumefantrine | *Standard Stock Preparation*: Transfer 20mg artemether and 120mg of lumefantrine to a 100mL flask. Add 15mL of dichloromethane and sonicate for 5mins. Make up to the mark with acetonitrile. *Sample Preparation:* Pulverize 20 tablets and weigh an equivalent of 20mg artemether and 120mg of lumefantrine into a 100mL flask. Add 15mL of dichloromethane and sonicate for 10mins. Make up to the mark with acetonitrile and sonicate for another 10min. Filter with a 0.45µm syringe filter and transfer filtrate to vial for HPLC analysis. | MP: o-phosphoric acid in water (pH:3.0±0.1)/acetonitrile (40:60) SP: Waters Xbridge C18 (100 x 4.6mm I.D, 5um) FR: 1.0mL/min, CT.: 40ᵒC, Inj. vol.: 10µL, λ: 210nm |
| Methyldopa | *Standard Preparation:* Dissolve methyldopa standard in methanol/glacial acetic acid (9.5:0.5). Sonicate for 10mins. Prepare calibration standards in mobile phase *Sample Preparation:* Pulverize 10 tablets and weigh an equivalent to 100mg Methydopa into a 100mL flask. Add 75mL of methanol/glacial acetic acid (9.5:0.5), sonicate for 20mins and make up to the mark with same extraction solvent. Transfer 1mL of the solution to a 10mL flask and make up to the mark with extraction solvent. Filter with a 0.45µm syringe filter and transfer filtrate to vial for HPLC analysis. | MP: 0.1%v/v trifluoroacetic acid in water/acetonitrile (87:13) SP: Phenomenex Luna C18 (250 x 4.6mm I.D, 5um) FR: 1.0mL/min, CT.: 35ᵒC, Inj. vol.: 10µL, λ: 286nm |
| Metronidazole | *Standard Preparation:* Dissolve metronidazole standard in mobile phase. Sonicate for 10mins. Prepare calibration standards in mobile phase. *Sample Preparation:* Pulverize 10 tablets and weigh an equivalent to 100mg metronidazole into a 100mL flask. Add 75mL of methanol, sonicate for 10mins and make up to the mark with same extraction solvent. Transfer 1mL of the solution to a 10mL flask and make up to the mark with mobile phase. Filter with a 0.45µm syringe filter and transfer filtrate to vial for HPLC analysis. | MP: Water/Methanol (80:20) SP: Waters Xterra C8 (150 x 4.6mm I.D, 5um) FR: 1.0mL/min, CT.: 40ᵒC, Inj. vol.: 10µL, λ: 254nm |
| Ciprofloxacin | *Standard Preparation:* Dissolve ciprofloxacin hydrochloride standard in mobile phase. Sonicate for 10mins. Prepare calibration standards in mobile phase A. *Sample Preparation:* Pulverize 20 tablets and weigh an equivalent to 100mg ciprofloxacin hydrochloride into a 100mL flask. Add 75mL mobile phase. Sonicate for 10mins and make up to the mark with same extraction solvent. Transfer 1mL of the solution to a 10mL flask and make up to the mark with extraction solution. Filter with a 0.45µm syringe filter and transfer filtrate to vial for HPLC analysis. | MP: o-phosphoric acid in water (pH:3.0±0.1)/acetonitrile (85:15) SP: Waters Xbridge C18 (100 x 4.6mm I.D, 5um) FR: 1.0mL/min, CT.: 40ᵒC, Inj. vol.: 10µL, λ: 278nm |
| Nifedipine | *Standard Preparation:* Dissolve Nifedipine standard in methanol. Sonicate for 10mins. Prepare calibration standards in mobile phase *Sample Preparation:* Pulverize 20 tablets and weigh an equivalent to 100mg Nifedipine into a 100mL flask. Add 75mL of methanol, sonicate for 10mins and make up to the mark with same extraction solvent. Transfer 1mL of the solution to a 10mL flask and make up to the mark with mobile phase. Filter with a 0.45µm syringe filter and transfer filtrate to vial for HPLC analysis. | MP: Water/Acetonitrile (45:55) SP: Waters Xbridge C18 (100 x 4.6mm I.D, 5um) FR: 1.0mL/min, CT.: 40ᵒC, Inj. vol.: 10µL, λ: 235nm |
| Ofloxacin and Tinidazole | *Standard Preparation:* Dissolve ofloxacin and tinidazole standard in extraction solvent (methanol/ glacial acetic acid (3:1)). Sonicate for 10mins. Prepare calibration standards in water/acetonitrile (9:1). *Sample Preparation:* Pulverize 10 tablets and weigh an equivalent to 40mg ofloxacin and 120mg tinidazole into a 100mL flask. Add 75mL of extraction solvent, sonicate for 20mins and make up to the mark with same extraction solvent. Transfer 1mL of the solution to a 10mL flask and make up to the mark with water/acetonitrile (9:1). Filter with a 0.45µm syringe filter and transfer filtrate to vial for HPLC analysis. | MP: 0.1%v/v trifluoroacetic acid in water/acetonitrile (80:20) SP: Phenomenex Luna C18 (250 x 4.6mm I.D, 5um) FR: 1.0mL/min, CT.: 35ᵒC, Inj. vol.: 20µL, λ: 303nm |
| Artemisinin | *Standard Stock Preparation:* Transfer 20mg artemisinin to a 20mL flask. Add 10mL of ethanol, sonicate for 10mins and make up to the mark with ethanol.  *Sample Preparation:* Pulverize 20 tablets and weigh an equivalent of 50mg artemisinin into a 100mL flask. Add 60mL of ethanol and sonicate for 10mins. Make up to the mark with ethanol and sonicate for another 10min. Filter with a 0.45µm syringe filter and transfer filtrate to vial for HPLC analysis. | MP: 0.1%v/v trifluoroacetic acid in water/acetonitrile (35:65) SP: Phenomenex Luna C18 (250 x 4.6mm I.D, 5um) FR: 1.0mL/min, CT.: 35ᵒC, Inj. vol.: 20µL, λ: 210nm |
| Piperaquine | *Standard Stock Preparation:* Transfer 100mg piperaquine to a 100mL flask. Add 15mL of dichloromethane, sonicate for 10mins and make up to the mark with acetonitrile. Prepare calibration standards in acetonitrile. *Sample Preparation:* Pulverize 20 tablets and weigh an equivalent of 100mg piperaquine into a 100mL flask. Add 15mL of dichloromethane and sonicate for 10mins. Make up to the mark with acetonitrile and sonicate for another 10min. Transfer 1mL of the sample solution into a 10mL flask and make up to the mark with acetonitrile. Filter with a 0.45µm syringe filter and transfer filtrate to vial for HPLC analysis. | MP: 0.1%v/v trifluoroacetic acid in water/acetonitrile (80:20) SP: Phenomenex Luna C18 (250 x 4.6mm I.D, 5um) FR: 1.0mL/min, CT.: 35ᵒC, Inj. vol.: 5µL, λ: 240nm |
| Pyrantel Pamoate | *Standard Preparation:* Dissolve pyrantel pamoate standard in methanol. Sonicate for 10mins. Prepare calibration standards in methanol. *Sample Preparation:* Pulverize 20 tablets and weigh an equivalent to 100mg pyrantel pamoate into a 100mL flask. Add 75mL of methanol, sonicate for 10mins and make up to the mark with same extraction solvent. Transfer 1mL of the solution to a 10mL flask and make up to the mark with methanol. Filter with a 0.45µm syringe filter and transfer filtrate to vial for HPLC analysis. | MP: 0.1%v/v trifluoroacetic acid in water/acetonitrile (80:20) SP: Phenomenex Luna C18 (250 x 4.6mm I.D, 5um) FR: 1.0mL/min, CT.: 35ᵒC, Inj. vol.: 10µL, λ: 286nm |
| Pyrimethamine and Sulfadoxine | *Standard Stock Preparation:* Transfer 400mg Sulfadoxine and 20mg of Pyrimethamine to a 100mL flask. Add 60mL of methanol and sonicate for 10mins. Make up to the mark with methanol. Prepare calibration standards in methanol. *Sample Preparation:* Pulverize 20 tablets and weigh an equivalent of 400mg Sulfadoxine and 20mg of Pyrimethamine into a 100mL flask. Add 60mL of methanol and sonicate for 10mins. Make up to the mark with methanol. Transfer 1mL of the sample solution into a 10mL flask and make up to the mark with methanol. Filter with a 0.45µm syringe filter and transfer filtrate to vial for HPLC analysis. | MP: 0.1%v/v phosphoric acid in water /acetonitrile (80:20) SP: Phenomenex Luna C18 (250 x 4.6mm I.D, 5um) FR: 1.0mL/min, CT.: 40ᵒC, Inj. vol.: 20µL, λ: 286nm |
