# Supplementary material for: A comparative study between Near-Infrared (NIR) spectrometer and High-Performance Liquid Chromatography (HPLC) on the sensitivity and specificity
Source: PLoS One. 2025 Mar 25;20(3):e0319523. doi: 10.1371/journal.pone.0319523 (PMC11936202; doi:10.1371/journal.pone.0319523)
Supplement: S3 Table — (DOCX) [file pone.0319523.s003.docx]

**S3 Table. Passing and failing rates by test and category of medicines**

|  | **HPLC** | | **NIR spectrometer** | |
| --- | --- | --- | --- | --- |
|  | Fail | Pass | Fail | Pass |
| **Analgesics** | 19 (17%) | 91 (83%) | 55 (50%) | 55 (50%) |
| **Antibiotics** | 12 (32%) | 26 (68%) | 0 | 38 (100%) |
| **Antihypertensives** | 22 (71%) | 9 (29%) | 0 | 31 (100%) |
| **Antimalarials** | 9 (13%) | 58 (87%) | 0 | 67 (100%) |
| **Total (N=246)** | 62 (25%) | 184 (75%) | 55 (22%) | 191 (78%) |
