# Supplementary material for: A comparative study between Near-Infrared (NIR) spectrometer and High-Performance Liquid Chromatography (HPLC) on the sensitivity and specificity
Source: PLoS One. 2025 Mar 25;20(3):e0319523. doi: 10.1371/journal.pone.0319523 (PMC11936202; doi:10.1371/journal.pone.0319523)
Supplement: S4 Table — (DOCX) [file pone.0319523.s004.docx]

**S4 Table. Sensitivity and Specificity of NIR spectrometer, by category of medicines.** N represents the number of drug samples in each cell.

**Panel A. Analgesics**

|  |  | **HLPC Lab** | |  |
| --- | --- | --- | --- | --- |
|  |  | Fail | Pass |  |
| **NIR spectrometer** | Fail | True positives (TP) | False positives (FP) |  |
|  |  | N=7 | N=48 | N=55 |
|  | Pass | False negatives (FN) | True negatives (TN) |  |
|  |  | N=12 | N=43 | N=55 |
|  | Total | N=19 | N=91 | N=110 |
| Sensitivity | TP/(TP+FN) |  |  | 37% |
| Specificity | TN/(TN+FP) |  |  | 47% |

**Panel B. Antibiotics**

|  |  | **HLPC Lab** | |  |
| --- | --- | --- | --- | --- |
|  |  | Fail | Pass |  |
| **NIR spectrometer** | Fail | True positives (TP) | False positives (FP) |  |
|  |  | N=0 | N=0 | N=55 |
|  | Pass | False negatives (FN) | True negatives (TN) |  |
|  |  | N=12 | N=26 | N=38 |
|  | Total | N=12 | N=26 | N=38 |
| Sensitivity | TP/(TP+FN) |  |  | 0% |
| Specificity | TN/(TN+FP) |  |  | 100% |

**Panel C. Antihypertensives**

|  |  | **HLPC Lab** | |  |
| --- | --- | --- | --- | --- |
|  |  | Fail | Pass |  |
| **NIR spectrometer** | Fail | True positives (TP) | False positives (FP) |  |
|  |  | N=0 | N=0 | N=55 |
|  | Pass | False negatives (FN) | True negatives (TN) |  |
|  |  | N=22 | N=9 | N=31 |
|  | Total | N=22 | N=9 | N=31 |
| Sensitivity | TP/(TP+FN) |  |  | 0% |
| Specificity | TN/(TN+FP) |  |  | 100% |

**Panel D. Antimalarials**

|  |  | **HLPC Lab** | |  |
| --- | --- | --- | --- | --- |
|  |  | Fail | Pass |  |
| **NIR spectrometer** | Fail | True positives (TP) | False positives (FP) |  |
|  |  | N=0 | N=0 | N=55 |
|  | Pass | False negatives (FN) | True negatives (TN) |  |
|  |  | N=9 | N=58 | N=67 |
|  | Total | N=9 | N=58 | N=67 |
| Sensitivity | TP/(TP+FN) |  |  | 0% |
| Specificity | TN/(TN+FP) |  |  | 100% |
