# Supplementary material for: A comparative study between Near-Infrared (NIR) spectrometer and High-Performance Liquid Chromatography (HPLC) on the sensitivity and specificity
Source: PLoS One. 2025 Mar 25;20(3):e0319523. doi: 10.1371/journal.pone.0319523 (PMC11936202; doi:10.1371/journal.pone.0319523)
Supplement: Attachment — Submitted filename: PONED2454221.docx [file pone.0319523.s005.docx]

PONED2454221

1) use of "counterfeit"

2) describe the library for the NIR
