# Supplementary material for: A comparative study between Near-Infrared (NIR) spectrometer and High-Performance Liquid Chromatography (HPLC) on the sensitivity and specificity
Source: PLoS One. 2025 Mar 25;20(3):e0319523. doi: 10.1371/journal.pone.0319523 (PMC11936202; doi:10.1371/journal.pone.0319523)
Supplement: Attachment — Submitted filename: PONE_responses_referees.docx [file pone.0319523.s007.docx]

**Additional Response to the Editor**

Journal Requirements:

When submitting your revision, we need you to address these additional requirements.

1. Please ensure that your manuscript meets PLOS ONE's style requirements, including those for file naming. The PLOS ONE style templates can be found at 
<https://journals.plos.org/plosone/s/file?id=wjVg/PLOSOne_formatting_sample_main_body.pdf> and 
<https://journals.plos.org/plosone/s/file?id=ba62/PLOSOne_formatting_sample_title_authors_affiliations.pdf>

Thank you very much for these suggestions. We adjusted the title page, abstract, references to table as per PLOSOne format.

2. Thank you for stating the following financial disclosure: The project was funded by USAID-DIV (7200AA21FA00006) and internal funding from the University of Michigan.
Please state what role the funders took in the study.  If the funders had no role, please state: ""The funders had no role in study design, data collection and analysis, decision to publish, or preparation of the manuscript."" 
If this statement is not correct you must amend it as needed. 
Please include this amended Role of Funder statement in your cover letter; we will change the online submission form on your behalf.

We added that funders had no role in the financial disclosure statement and in the cover letter.

3. In the online submission form, you indicated that the datasets used and/or analyzed during the current study are available from the corresponding author on reasonable request.
All PLOS journals now require all data underlying the findings described in their manuscript to be freely available to other researchers, either 1. In a public repository, 2. Within the manuscript itself, or 3. Uploaded as supplementary information.
This policy applies to all data except where public deposition would breach compliance with the protocol approved by your research ethics board. If your data cannot be made publicly available for ethical or legal reasons (e.g., public availability would compromise patient privacy), please explain your reasons on resubmission and your exemption request will be escalated for approval.

We uploaded the data on lab test results and scanner test for N=246 as Supplementary Information. The dataset contains category of drug, brand, batch number, expiration data, API found from HPLC, whether the drug sample passed HPLC or pass NIR, city, LGA, rural/urban.

4. Please include your full ethics statement in the ‘Methods’ section of your manuscript file. In your statement, please include the full name of the IRB or ethics committee who approved or waived your study, as well as whether or not you obtained informed written or verbal consent. If consent was waived for your study, please include this information in your statement as well.

5. Please include captions for your Supporting Information files at the end of your manuscript, and update any in-text citations to match accordingly. Please see our Supporting Information guidelines for more information: <http://journals.plos.org/plosone/s/supporting-information>.

**Response to Reviewers**

**Reviewer #1:** The paper submitted by Maffioli and Anyakora discusses the « validation » of a NIR spectrometer to detect SF medicines.
As mentioned by the authors, rigorous and transparent validation of screening devices such as NIR handheld spectrometers is mandatory before using it in routine.
However, the paper is anything but transparent. No information is given on the device used, the algorithms used, how the models have been trained and no detail is given on the obtained results. Therefore, it is impossible to understand what the limitations of this technique are and what may be improved. This paper, in its present form, cannot be published since it does not add any new information and may be prejudicial to the reputation of promising techniques such as NIR spectroscopy. Indeed, each technology comes with its own advantages and drawbacks. This is why is it imperative to explain what the objective of the models was and what the results are.

We apologize that some details are missing. This is primarily due to the lack of transparency by the company, which is in fact a key learning lesson mentioned in the discussion of the limitations as follows: *Second, due to the proprietary nature of the machine-learning algorithm and the hardware and software, and to protect proprietary information and sensitive business data, we are unable to disclose the company’s identity. This constraint limits the researchers’ ability to fully share technical details and methodologies related to the device's design, calibration, and performance metrics.*

This is also discussed in the conclusion: *Our study underscores the need for greater transparency from manufacturers, particularly regarding their data analytics methods and the processes they employ. Without this transparency, it remains challenging to assess the reliability and accuracy of such devices in screening SF medicines.*

We now revised the Methods to include more detailed information we have from the company on technical capabilities of the device and process for library construction. Please see responses to your next comments.

Detailed comments:

- L111: what do the authors mean when they say that handheld devices are not field ready? The objective of these devices is to be used in the field, and they are efficiently used in many field settings since a long time.

Thank you for pointing this out. We meant that they had not undergone exhaustive testing to be ready for deployment by policymakers. However, we agree that this statement may not apply to all handheld spectrometers. We have now revised the sentence to state that they are costly and heavy.

- L112: what do the authors mean by “low-cost”? This appears subjective.

Related to the point above, we note that, given the high cost of handheld spectrometers, portable screening devices that are less expensive are being developed. We now use the term 'lower-cost' (compared to what was mentioned earlier) and have added a citation to Vickers et al. (2018), which compares the costs of these devices and confirms that portable devices are indeed less expensive than handheld spectrometers. This is further described in the Methods section, where we discuss the cost of the NIR tested.

- L121: what is a “validation study”? The present article does not follow any validation guidelines. Please refer to the existing guidelines for validation or do not use this terminology.

Thank you very much for raising this point. Based on your comments, as well as similar feedback from the other referee, we agree that we are not conducting a validation of the NIR and that the scope is limited. We have now updated the title to *'A Comparative Study Between Near-Infrared (NIR) Spectrometer and High-Performance Liquid Chromatography (HPLC) on Sensitivity and Specificity.'* We have also adjusted the text accordingly, removing the term ‘validation study’ and instead referring to it as a ‘comparative study’ or ‘exercise’.

- The anonymization of the whole NIR part is not acceptable since it is the core of the paper. It is impossible to the reviewer to understand the few information provided. Where the measures performed in reflectance or transmittance? What is the kind of detector, how is performed the white reference, … It is mentioned that the device is “similar to NIR-S-G1” but the spectral range is completely different… In which sense is it similar? Regarding the algorithms used, just calling it “AI” is insulting for the reader. What is this? Is it a regression approach to quantify the API? Is it a qualitative approach to authenticate a brand or to identify an API? How are the algorithms trained? How have they been validated? This is really the core of the device and no information at all is provided.

We apologize for being unable to provide more details on the NIR due to the anonymization. Following your and other comments of the reviewers we added further details on the library construction which we hope will help addressing some of your concerns.

*…Customized chemometric models are necessary to develop the reference library, with authentic samples sourced for a fee by the company. The company claimed at the time that any product (in pill form) could be analyzed with their NIR device, provided the reference library was updated. For this specific study, the company was responsible for sourcing the exact branded drug samples and dosage forms (though not the lot numbers tested in the field). [footnote 2] Unlike HPLC, this portable device does not require sample destruction and enables real-time analysis, making it accessible for regulators, law enforcement, customs, manufacturers, and pharmacies…*

*Footnote 2: More specifically, the company stated that 3 out of the 20 drugs in our sample were already included in their library: May & Baker Para (Paracetamol) Tabs. (x96), Emzor Paracetamol Tabs., 500mg, and Lonart-DS Artemether Lumefantrine Tabs., 80mg/480mg. The remaining drug samples were sourced and added to the reference library by the company. The company did not share the results of their training exercises.*

We also added details on how the NIR scanner provides a match/non-match results as follows:

*…The spectrometer analyzes a drug’s spectral signature to detect poor-quality medicines by comparing it to a cloud-based AI reference library of spectral signatures. The NIR is capable of detecting both substandard and counterfeit drugs. The device captures the spectrum of the entire drug (API and excipients) and stores the spectral signature of this medical product. It also captures the intensity of the spectrum, which is proportional to that of the authentic product from the manufacturer. The device can determine counterfeit drug samples by matching the signature spectrum of the reference product with the drug sample collected in the field. If the spectrum of the pill collected in the field differs from the spectrum of the authentic sample, the spectrometer displays a “non-match” result. The device is also able to detect substandard drug samples by matching the intensity of the reference product with the drug sample collected in the field. Researchers were not given details on other parameters or thresholds, which may vary by drug. The process takes about 20 seconds, with a quality report sent to a smartphone app...*

We agree that the statement “similar to NIR-S-G1” is very vague and we cannot speak to all the details you are asking to compare. We decided to remove old footnote 1.

- There is no detail on the dataset. Only 256 spectra are mentioned in the tables but at L186, 1142 samples have been tested with NIR. Where are the remaining 886 spectra?

Thank you for raising this point. Our comparative study focuses on a sub-sample of 246 drugs that were tested with both NIR and HPLC. However, the referee is correct that the rest of the sample was tested only with NIR. Unfortunately, due to resource constraints, we were unable to test all drug samples with HPLC as well. We do not believe that the rest of the sample is relevant to this comparative study.

For the referee’s information, we note that all other categories of drugs, except for analgesics, yielded a 'match' result with NIR. Additionally, the mobile phone app associated with the NIR did not automatically record all spectra. We were able to recover a sub-sample of these spectra from some of the devices used for NIR testing, and we are happy to share this data if it would be of interest for further analysis. However, we would be unable to compare these additional drug samples with HPLC, as they were not tested in the laboratory.

As suggested by you and other referees, we have included the data for the 246 drug samples tested with HPLC in the Supplementary Information. The dataset contains category of drug, brand, batch number, expiration data, API found from HPLC, whether the drug sample passed HPLC or pass NIR, city, LGA, rural/urban.

- The dataset is briefly described but once again no details. How many samples are collected per brand? Are there different batches?

The 20 drug types tested were branded drugs, and we collected samples for each branded drug from the set of 20. We have now added the number of samples tested for each of these drugs in Table 1.

Since the drug samples were purchased by 12 mystery shoppers across 6 geopolitical locations, with only one drug sample collected per pharmacy visited, the samples are likely from different batches. Our data confirms this. Below, we provide a table showing the duplicate batch numbers: d |	Freq. Percent	Cum.

------------+-----------------------------------

0 |	163	66.26	66.26

1 |	32	13.01	79.27

2 |	24	9.76	89.02

3 |	4	1.63	90.65

Missing |	23	9.35	100.00

------------+-----------------------------------

Total |	246	100.00

Note that for 23 drug samples, the enumerator was unable to observe the batch number on the package. Of the samples with batch numbers, 163 out of 223 (73%) are from distinct batches. Two batch numbers are repeated twice, eight batch numbers are repeated three times, and 16 batch numbers are repeated twice.

- The HPLC results should also be provided. 62 samples where outside the 90-110% range. But how far? 89% or 25% is a totally different story. NIR in reflectance on finished product is not a magic tool. The results will depend on how the system is calibrated. Once again, we have no information.

Thank you for your comment. We describe partly some of these results in Maffioli et al. 2024, where we describe the proportion of the drug samples who failed because of low or high APIs. See details here.

*We present evidence from an ongoing research study in Nigeria, as part of which we tested 246 medicine samples, including analgesics (45%), antimalarials (27%), antibiotics (15%), and antihypertensives (13%). Among all medicines, 62 (25%) were found to have API outside the range (90–110%) as tested by high-performance liquid chromatography, suggesting that the prevalence of SF medicines is still quite high in the country. Among those who did not pass the laboratory testing, 35% (n=22) were antihypertensives, 31% (n=19) were analgesics, 19% were antibiotics (n=12), and 15% (n=9) were antimalarials. Most of these drugs (76%, n = 47) had two APIs. Further, 65% (n = 40) of drugs failed because at least one (or only one) ingredient had an API exceeding the limit. Among these, 50% (n = 31) of medicines with two ingredients failed because one was below and one was above the range, and 15% (n = 9) failed because one or two APIs in the medicines exceeded the 110% limit. The rest (35%, n = 22) failed because of their API being too low (Table 1).*

More importantly, in response to your comment, we have now provided the data in the Supplementary Material (n=246). Among the samples that failed due to low API—addressing the concerns you raised—21 drug samples have at least one API lower than 50%. Specifically, around 30–40% of amiloride was found in amiloride+hydrochlorothiazide tablets (Normoretic or Bonduretic antihypertensives). Please refer to the data for detailed results on each sample

- No information is provided on the fact that the API was correctly present or not. Where all samples containing the correct API?

We hope the description in Maffioli et al. 2024 and the data above address this point.

- The detection of substandards imply the quantitation of the samples. How was this performed? NIR only “sees” API/excipients ratios. Therefore, have the masses of the tablets been measured?

We apologize for missing the details here. We now added more information in the text as mentioned above in one of your previous comments and here as follows:

*…The spectrometer analyzes a drug’s spectral signature to detect poor-quality medicines by comparing it to a cloud-based AI reference library of spectral signatures. The NIR is capable of detecting both substandard and counterfeit drugs. The device captures the spectrum of the entire drug (API and excipients) and stores the spectral signature of this medical product. It also captures the intensity of the spectrum, which is proportional to that of the authentic product from the manufacturer. The device can determine counterfeit drug samples by matching the signature spectrum of the reference product with the drug sample collected in the field. If the spectrum of the pill collected in the field differs from the spectrum of the authentic sample, the spectrometer displays a “non-match” result. The device is also able to detect substandard drug samples by matching the intensity of the reference product with the drug sample collected in the field. Researchers were not given details on other parameters or thresholds, which may vary by drug. The process takes about 20 seconds, with a quality report sent to a smartphone app...*

**Reviewer #2:** The authors compared the quality of 246 medicines using an inexpensive handheld spectrophotometer and a gold standard method, HPLC. The medicines were collected in Nigeria. While the finding that about 25% of the products failed HPLC assay is not unexpected given the wealth of previous research about poor quality medicines in LMICs including Nigeria, this manuscript highlights the pitfalls of the currently unregulated market for devices like the NIR spectrophotometer, that claim to detect substandard or falsified pharmaceuticals (SFPs). The prior literature is treated fairly and the writing is concise and well organized. I do have some concerns about the manuscript, described below, but I think the paper provides very useful information that could save other researchers and regulators a lot of trouble in future efforts to detect SFPs. I hope the authors revise and resubmit.

Dear Prof. Lieberman,

We very much appreciate your feedback, and we hope the changes made to the manuscript address your concerns and improve the description of the findings. Thanks a lot.

The methodology is described clearly, with one exception: it's not clear what discussions the authors had with the manufacturer about the product's training/library, or what the unnamed manufacturer claimed about their product's capabilities.

A1. The method section says that the company "produces a customized reference library with authentic samples sourced for a fee".
-->describe what samples went into the reference library that was used in this study. To be specific, was the library trained with the same exact brands/lot numbers as the samples that were tested in the field? Did the company do any testing to make sure their training samples were actually good quality? That's been a problem in previous studies. If not the same brands, were they the same dosage forms (eg, tablets with same dose of API)? For samples like the analgesics where several brands contained multiple drugs, were correct reference samples used in the NIR library?
-->If it's not known what samples were in the reference library (eg, because the company will not disclose) that needs to be stated, along with any claims the company makes about what products can be analyzed with their NIR.

We apologize for not providing more details on the library construction. Given the version of the device used, the company claimed at the time that any product (in pill form) could be analyzed with their NIR device, provided the reference library was updated. For this specific study, the company was responsible for sourcing the exact branded drug samples and dosage forms (though not the lot numbers tested in the field) and for training the model.

More specifically, the company stated that 3 out of the 20 drugs in our sample were already included in their library: May & Baker Para (Paracetamol) Tabs. (x96), Emzor Paracetamol Tabs., 500mg, and Lonart-DS Artemether Lumefantrine Tabs., 80mg/480mg. The remaining drug samples were sourced and added to the reference library by the company. However, the company did not share the results of their training exercise for either the drug samples already in the library or those added for this study.

As such, we now acknowledge in the discussion that this lack of transparency could potentially be a source of error or limitation in the study.

*…Third, building and maintaining an up to date "spectral reference library" with quality-assured genuine samples is both challenging and costly. Authentic materials are often difficult to obtain and may not be suitable for screening finished products, as spectra can be influenced by both APIs and excipients and can vary between brands (Vickers et al. 2018). In our study, the company was responsible for building a customized reference library using authentic samples procured directly from the manufactures. The researchers did not have control over this process, and the company did not share the results of their procedure. As a result, we cannot be certain that the agreed-upon process was followed, although we have no reason to believe otherwise. Our findings suggested that the NIR spectrometer’s AI algorithm was better trained on analgesics, highlighting the need for a comprehensive and shareable "spectral reference library” to facilitate effective device training and comparison.*

A2. In the Methods section, the authors state that the non-match result from the NIR is a result of "alternative excipients, APIs, or contaminants". However, the HPLC results do not show that any of the products contained alternative excipients, APIs, or contaminants. The HPLC results are just assays.
--> Do the manufacturers actually make the claim that the NIR spectrophotometer can detect substandard products? If not, there needs to be some discussion of why the authors are calling it a failure of the instrument when it missed substandard drugs. I think they could make this argument even if the mfr does not make the claim, as it is a serious problem if the NIR cannot detect a product with very low API content, eg <50%, but there should be some acknowledgement that "s" and "f" products are different challenges.

Thank you for raising this very important point. We apologize for the confusion, and we now removed that specific sentence. As you correctly stated, we agree that HPLC does not show excipients or contaminants, but the test is only able to identify whether the API are outside the range. The company does make the claim that NIR can detect substandard drugs. We now revised the methods as follows:

*…The spectrometer analyzes a drug’s spectral signature to detect poor-quality medicines by comparing it to a cloud-based AI reference library of spectral signatures. The NIR is capable of detecting both substandard and counterfeit drugs. The device captures the spectrum of the entire drug (API and excipients) and stores the spectral signature of this medical product. It also captures the intensity of the spectrum, which is proportional to that of the authentic product from the manufacturer. The device can determine counterfeit drug samples by matching the signature spectrum of the reference product with the drug sample collected in the field. If the spectrum of the pill collected in the field differs from the spectrum of the authentic sample, the spectrometer displays a “non-match” result. The device is also able to detect substandard drug samples by matching the intensity of the reference product with the drug sample collected in the field. Researchers were not given details on other parameters or thresholds, which may vary by drug. The process takes about 20 seconds, with a quality report sent to a smartphone app.*

*Customized chemometric models are necessary to develop the reference library, with authentic samples sourced for a fee by the company. The company claimed at the time that any product (in pill form) could be analyzed with their NIR device, provided the reference library was updated. For this specific study, the company was responsible for sourcing the exact branded drug samples and dosage forms (though not the lot numbers tested in the field). [footnote 2 Unlike HPLC, this portable device does not require sample destruction and enables real-time analysis, making it accessible for regulators, law enforcement, customs, manufacturers, and pharmacies…*

*Footnote 2: More specifically, the company stated that 3 out of the 20 drugs in our sample were already included in their library: May & Baker Para (Paracetamol) Tabs. (x96), Emzor Paracetamol Tabs., 500mg, and Lonart-DS Artemether Lumefantrine Tabs., 80mg/480mg. The remaining drug samples were sourced and added to the reference library by the company. The company did not share the results of their training exercises.*

To add to your last point about API content, we describe partly some of these results in Maffioli et al. 2024, where we present the proportion of the drug samples who failed because of low or high APIs, as follows:

*We present evidence from an ongoing research study in Nigeria, as part of which we tested 246 medicine samples, including analgesics (45%), antimalarials (27%), antibiotics (15%), and antihypertensives (13%). Among all medicines, 62 (25%) were found to have API outside the range (90–110%) as tested by high-performance liquid chromatography, suggesting that the prevalence of SF medicines is still quite high in the country. Among those who did not pass the laboratory testing, 35% (n=22) were antihypertensives, 31% (n=19) were analgesics, 19% were antibiotics (n=12), and 15% (n=9) were antimalarials. Most of these drugs (76%, n = 47) had two APIs. Further, 65% (n = 40) of drugs failed because at least one (or only one) ingredient had an API exceeding the limit. Among these, 50% (n = 31) of medicines with two ingredients failed because one was below and one was above the range, and 15% (n = 9) failed because one or two APIs in the medicines exceeded the 110% limit. The rest (35%, n = 22) failed because of their API being too low (Table 1).*

More importantly, in response to your comment, we have now provided the data in the Supplementary Material (n=246). Among the samples that failed due to low API—addressing the concerns you raised—21 drug samples have at least one API lower than 50%. Specifically, around 30–40% of amiloride was found in amiloride+hydrochlorothiazide tablets (Normoretic or Bonduretic antihypertensives). Please refer to the data for detailed results on each sample.

B. The HPLC results are presented only in aggregate (eg, 50% of the analgesics failed to meet the HPLC content standard, but it's not clear which products failed or how badly they failed).
-->Please provide data on the individual products in the supporting information section. It would be ideal to list the full product metadata (brand, lot number, expiration date, API content found, NIR result). A standard circumlocution which protects the researcher is to say that the product is "stated to be Brand Wonderful, lot number 12345"--in this case, you are just reporting the information that is printed on the packet or blister pack. -->If the brand and lot number metadata cannot be disclosed, that should be explained (eg, "due to pending regulatory activity we were asked not to disclose brand names"). In this case, the brands could be coded as Brand A, B, C, etc, and expiration dates, API contents, and NIR results could be provided.

Thank you so much for suggesting this. We have now provided the data in the Supplementary Material (n=246). The dataset contains category of drug, brand, batch number, expiration data, API found from HPLC, whether the drug sample passed HPLC or pass NIR, city, LGA, rural/urban.

C. It is not stated whether the chemical analysis results for failing products (the HPLC, not the NIR) were reported to the applicable regulatory agency, which seems to me to be ethically necessary. This could be mentioned in the "methods" section
-->please report whether data about products that failed HPLC assay was shared with NAFDAC and/or WHO.

Thank you for raising this issue. As the drug samples were only collected for the purpose of being analyzed for this study, there was no risk associated with those samples who failed as they were not put back in the supply. However, we plan to report these samples to NAFDAC. NAFDAC is also currently doing their own testing of the same NIR device.

There are some minor comments for the authors:

D1. In the introduction, the term "counterfeit" is thrown around a lot. However, counterfeit does not mean "bad quality", any more than "authentic" means good quality--there are counterfeit versions of pharmaceuticals that are nearly identical to the original product, and there are a lot of products that were produced by their stated manufacturer that have dreadful quality, eg substandard amount of API, the wrong API, or wrong excipients. Since neither HPLC nor NIR claim to detect counterfeits anyway, can you just take it out?
-->Please rewrite that third paragraph of the intro, using "substandard and falsified" or "bad quality".

This is a very important point. Thank you for this suggestion. We adjusted the text in the introduction.

D2. The "Food and Drug Administration 1994" reference doesn't seem like the greatest reference for the value of NIR since it is 30 years old--instruments and data analytics have advanced a lot since the 90's!-->find a more current reference for the value of NIR in pharma screening

Thank you for pointing this out. The reference indeed pertains to the HPLC method rather than the statement in which it was cited. We have removed the reference accordingly. Since we are describing the portable device investigated in this study, we believe no reference is necessary for the statement, as it merely outlines the device's capabilities and limitations.

D3. table A3--header says HPCL, should be HPLC.

Thank for finding this typo. This was adjusted.

**Reviewer #3:** Title and content: “Validation of Near-Infrared Spectrometer to detect Substandard and Falsified Medicines”. This is confusing, and it doesn’t match the content. The title should be defined in the way it matches the content and defines the goal and scope of the study. Line 173-180 show that you have compared results by two different analytical techniques/equipments. In my understanding, you should target to validate an analytical method, and consider the method validation parameters as defined by ICH. In case you were interested in comparing the two analytical techniques/equipments, you should formulate the title appropriately and avoid the word “validation”. Therefore, define a title that matches the content of your work and findings. Even if we assume that the NIR apparatus corresponds to an analytical method, you shouldn’t claim to have validated it while you have considered only two parameters! You have done a good work, but it needs an appropriate title. Another issue is the comparison of a qualitative analytical technique (NIR-AI) to a quantitative analytical technique (HPLC): the NIR-AI is designed for the screening step, to detect deviations from the specific original/genuine formulations; but, HPLC quantifies the content of an specific active pharmaceutical ingredients using specific validated analytical methods. Suggested title: A comparative study between NIR and HPLC on the specificity and selectivity.

Dear Prof. Bizimana,

Thank you very much for raising this valid point. We very much appreciate your feedback and we agree with it. Following your suggestion regarding the title, we have made the following change: "A Comparative Study Between Near-Infrared (NIR) Spectrometer and High-Performance Liquid Chromatography (HPLC) on Specificity and Sensitivity."

Line 93-95: “This issue is especially prevalent in LMICs, where high prices limit access to authentic medicines, and inadequate government oversight allows counterfeiters to go unpunished”. This statement requires at least one supporting reference, you can’t just say this.

Thank you for your suggestions. We added Almuzaini et al. 2013 review which shows “that the prevalence of substandard/counterfeit antimicrobials reported was significantly higher in the unauthorised market”.

- Almuzaini, T., Choonara, I. and Sammons, H., 2013. Substandard and counterfeit medicines: a systematic review of the literature. BMJ open, 3(8), p.e002923.

We also added Abdoulaye et al. 2003 confirming unofficial sale of drugs in a LMIC is a common practice and considered a serious public health problem.

- Abdoulaye I, Chastanier H, Azondekon A. Survey on the illicit drug market in Cotonou, Benin in March 2003. Med Trop (Mars) 2006;66:573–6.

We also added Oldfield, Lachlan et al. 2024. showing that “medicines consistently display high prices, low affordability, and poor availability in 54 LMICs.”

- Oldfield, Lachlan et al. 2024. Prices, availability, and affordability of adult medicines in 54 low-income and middle-income countries: evidence based on a secondary analysis The Lancet Global Health, Volume 13, Issue 1, e50 - e58

Line 139-142: “Unlike HPLC, this portable device does not require sample destruction and enables real-time analysis, making it accessible for regulators, law enforcement, customs, manufacturers, and pharmacies (Food and Drug Administration, 1994)”. This reference doesn’t support this statement. You should also avoid references older than 10 years.

Thank you for pointing this out. The reference indeed pertains to the HPLC method rather than the statement in which it was cited. We have removed the reference accordingly. Since we are describing the portable device investigated in this study, we believe no reference is needed for the statement, as it merely outlines the device's capabilities and limitations.

Line 191-193: “Of those failing the HPLC test, 35% (N=22) were antihypertensives, 31% (N=19) were analgesics, 19% (N=12) were antibiotics, and 15% (N=9) were antimalarials (Maffioli et al. 2024)”. Unless this manuscript has been already published elsewhere, otherwise this reference should not be used to support your findings.

Thank you. This commentary was published in the Journal of Global Health. We describe partly some of these results in Maffioli et al. 2024, where we describe the proportion of the drug samples who failed because of low or high APIs. See details here.

*We present evidence from an ongoing research study in Nigeria, as part of which we tested 246 medicine samples, including analgesics (45%), antimalarials (27%), antibiotics (15%), and antihypertensives (13%). Among all medicines, 62 (25%) were found to have API outside the range (90–110%) as tested by high-performance liquid chromatography, suggesting that the prevalence of SF medicines is still quite high in the country. Among those who did not pass the laboratory testing, 35% (n=22) were antihypertensives, 31% (n=19) were analgesics, 19% were antibiotics (n=12), and 15% (n=9) were antimalarials. Most of these drugs (76%, n = 47) had two APIs. Further, 65% (n = 40) of drugs failed because at least one (or only one) ingredient had an API exceeding the limit. Among these, 50% (n = 31) of medicines with two ingredients failed because one was below and one was above the range, and 15% (n = 9) failed because one or two APIs in the medicines exceeded the 110% limit. The rest (35%, n = 22) failed because of their API being too low (Table 1).*

Conclusion (line 265-281): it is too much general. Kindly formulate a conclusion bound to your data/findings, keeping in mind the scope of your study. End the conclusion pointing out the identified gaps and suggesting interventions to address/fill in the gaps.

Thank you so much for pointing this out. We have revised the conclusion to clearly highlight the following gaps: (1) Transparency from manufacturers, and (2) Testing device effectiveness before deployment to protect patients. We also suggest ways to address these gaps: (1) Establish clear guidelines and foster collaborations, and (2) Increase field testing. The revised conclusion is as follows:

*Currently, no device has demonstrated the ability to screen the quality of all APIs across the wide range of formulations and settings required in real-world scenarios (Vickers et al. 2018). Our study underscores the need for greater transparency from manufacturers, particularly regarding their data analytics methods and the processes they employ. Without this transparency, it remains challenging to assess the reliability and accuracy of such devices in screening SF medicines. These findings reveal significant gaps in the current regulatory landscape for devices like the NIR spectrometer, which claim to detect SF pharmaceuticals. Given the limitations and lack of standardization in existing devices, regulatory bodies should consider establishing clear guidelines for device performance and testing in real-world settings. This would ensure that devices meet robust standards of accuracy and reliability before being marketed as solutions for screening SF medicines. Additionally, policymakers should encourage collaboration between regulatory authorities, manufacturers, and researchers to develop and validate reliable technologies for screening pharmaceuticals.*

*While these devices hold potential to be cost-effective and user-friendly, their effectiveness in protecting patients from SF medicines must remain the foremost priority. Devices with low sensitivity should undergo additional lab evaluations before being deployed in real-world settings. More generally, field testing remains essential, as several manufacturers continue to market NIR spectrometers based on limited laboratory test sets, without comprehensive field testing. Ultimately, ongoing research and testing are crucial to build a robust body of evidence that can guide regulators and other stakeholders in selecting the most effective tools to combat poor-quality medicines. Only through rigorous, transparent and continuous field testing and research can we ensure that these screening devices fulfill their promise of enhancing patient safety and strengthening the pharmaceutical supply chain.*
